# *Cimicifuga foetida* L. polysaccharide alleviates ulcerative colitis by inhibiting pyroptosis and regulating gut microbiota

**DOI:** 10.3389/fphar.2026.1780518

**Published:** 2026-07-13

**Authors:** Qing Luo, Yangyang Xu, Zhihua Li, Ximin Wang, Liu Cao, Gengting Dong, Shunan Guo, Weibo Dai

**Affiliations:** 1 Pharmacology Laboratory, Zhongshan Hospital of Traditional Chinese Medicine Affiliated to Guangzhou University of Traditional Chinese Medicine, Zhongshan, China; 2 State Key Laboratory of Mechanism and Quality of Chinese Medicine, Macau University of Science and Technology, Taipa, China

**Keywords:** Cimicifuga foetida L., gut microbiota, polysaccharide, pyroptosis, ulcerative colitis

## Abstract

**Introduction:**

*Cimicifuga foetida* L. is widely applied in the clinical treatment of ulcerative colitis (UC); however, its active components and mechanisms have not been deeply investigated. The objective of this study is to investigate the potential bioactive constituents of *C. foetida* L. for the treatment of UC, and elucidate its therapeutic mechanism.

**Methods:**

The crude polysaccharide of *C. foetida* L. was extracted by hot water and purified by DEAE Sepharose™ Fast Flow column to obtain, and named SM05. The structure was determined by HPLC, FT-IR and SEM. The effect of polysaccharide (SM05) on the mouse UC model and its mechanism of action were investigated using a dextran sodium sulfate (DSS)-induced UC model. Changes in body weight, disease activity index, colon length, organ index, histopathological injury, cytokine expression and intestinal tight junction proteins were measured to evaluate the effect of SM05 on UC. IHC, RT-qPCR, and 16s rDNA sequencing were performed to elucidate the underlying mechanism.

**Results:**

SM05 is mainly composed of mannose, glucose, galactose, and arabinose. SM05 exerts antioxidant effects by activating the Nrf2/Keap1 pathway, thereby inhibiting the NLRP3-induced pyroptosis pathway. This reduces abnormal intestinal cell death and the secretion of inflammatory cytokines, thus protecting the intestinal barrier and suppressing further inflammation. Additionally, SM05 modulates the gut microbiota structure in mice with ulcerative colitis by reducing pathogenic bacteria (e.g., *Bacteroides* and Desulfovibrio) that damage the intestinal barrier and increasing the abundance of beneficial bacteria (e.g., Akkermansia and Saccharibacteria), thereby alleviating the progression of ulcerative colitis.

**Discussion:**

In the preliminary biological assessment, the polysaccharide subfraction SM05 alleviated the symptoms of UC induced by DSS. This effect may be related to the activation of the Nrf2/Keap1 pathway, the inhibition of pyroptosis, and the regulation of the intestinal microbiota.

## 1 Introduction

Ulcerative colitis (UC) is an inflammatory bowel disease that occurs in the colorectum (Sadik et al., 2022). The pathogenesis of UC remains incompletely understood, and existing therapeutic options are limited (Dong et al., 2022). Therefore, exploring novel therapeutic strategies that are both effective and safe is a priority. Mounting evidence indicates that the development and progression of UC are closely associated with pyroptosis and gut microbiota dysbiosis (Zhang et al., 2024).

Pyroptosis is a form of caspase-1-dependent programmed cell death mediated by the NLRP3 inflammasome. This process exacerbates intestinal inflammation through the release of pro-inflammatory cytokines IL-1β and IL-18, thereby disrupting mucosal integrity (Gu et al., 2022). Furthermore, disruption of gut microbiota balance impairs intestinal defense and immune regulatory functions (Shen et al., 2018). Notably, specific pathogenic bacteria, such as *Proteus mirabilis*, can directly activate the NLRP3 inflammasome within intestinal mucosal mononuclear phagocytes (Watanabe et al., 2021). Therefore, a therapeutic strategy that simultaneously inhibits the NLRP3-mediated pyroptosis pathway and modulates gut microbiota composition represents a promising approach for UC treatment.

*Cimicifuga foetida* L*.* is a perennial herb belonging to the genus Cimicifuga of the Ranunculaceae. *Cimicifuga foetida* L. has been used since ancient times both as a functional food and as an herbal remedy in various traditional medical systems worldwide, owing to its anti-inflammatory, antipyretic, and analgesic properties (Cao et al., 2005; Siddiqui et al., 2022). In traditional Chinese medicine (TCM) clinical practice, formulations such as Shengma Gegen Tang and Huchang Wan are commonly used to treat UC, with *C. foetida* L. being a key medicinal component. Pharmacological studies have shown that *C. foetida* L. extract can alleviate inflammation and oxidative stress through inhibition of the Nrf2/HO-1/NQO1 signaling pathway (Lyu et al., 2006; Lim and Song et al., 2021). However, the mechanism underlying the anti-UC effect of polysaccharide components derived from Cimicifuga foetida L. has not been reported in the literature.

In our study, we isolated a polysaccharide subfraction named SM05 with anti-UC activity from *C. foetida* L. and performed a preliminary characterization of its structure. Subsequently, the mechanism of SM05’s anti-UC effects was investigated using pyroptosis and intestinal microbiota as perspectives.

## 2 Materials and methods

### 2.1 Preparation of polysaccharides

Take 1 kg of the *C. foetida* L. (ZhiXin Traditional Chinese Medicine Slices Co., Ltd., Guizhou, China, Lot No. 221201), and extract it twice with 20 L of purified water, with each extraction lasting for 2 h. All water-extracted solutions were collected together and concentrated. After centrifugation (4,000 rpm × 15 min), the supernatant was placed in a 3500 Da dialysis bag and dialyzed with tap water for 12 h. The solution should be concentrated in the bag and centrifuged at 4,000 rpm for 15 min. Finally, the supernatants were precipitated by adding 1:3 (v/v) 95% ethanol overnight, and the precipitate was isolated and freeze-drying to obtain the crude polysaccharide (SMW). The crude polysaccharide SMW was dissolved in ultrapure water at a ratio of 1:10 (m/v) and stirred on a magnetic stirrer for 12 h. The mixture was centrifuged at 3,434 *g* for 15 min, and the supernatant was collected. Gradient elution was performed using 0, 0.05, 0.1, 0.2, 0.4, 0.8, and 1 mol/L NaCl solutions, and the separation was carried out on a DEAE Sepharose™ Fast Flow column to obtain various secondary polysaccharides. The mobile phase flow rate was 1 mL/min (15 mL per tube). An aliquot of 20 µL from each tube was mixed with 40 µL of 5% phenol aqueous solution and 200 µL of concentrated sulfuric acid. After 15 min, the absorbance was measured at 490 nm to generate the elution curve. The fractions corresponding to the absorption peak of the 0.05 mol/L NaCl elution were pooled, concentrated under reduced pressure, dialyzed, and lyophilized to obtain the secondary polysaccharide SM05 from *C. foetida* L.

### 2.2 Determination of the physicochemical properties of polysaccharides

The neutral sugars content of SM05 was measured by phenol-sulfuric acid method using glucose as a standard. Using glucuronic acid as standard, the content of glucuronic acid was determined by m-phenylphenol method. Protein content was measured according to the BCA method, using bovine serum albumin (Thermo Fisher Scientific, MA, United States, Cat. No. 23225) as the standard protein (Xu et al., 2024).

### 2.3 Molecular weight analysis

The weight-average molar mass (Mw) of SM05 was determined by high-performance gel permeation chromatography (HPGPC) using a Shimadzu LC-10A (Shimadzu, Kyoto, Japan) system equipped with a BRT105-103–101 column (8 × 300 mm) maintained at 40 °C. The analysis was performed using 0.05 M NaCl as the mobile phase at a flow rate of 0.8 mL/min. Mw was determined with reference to a calibration curve using standard dextran (1.153, 6.941, 9.835, 20.6, 47.88, 112.2, 240.1, 396.4, 883.1 and 1,689.32 kDa) plotted by linear regression (y = −0.222x + 11.44 R^2^ = 0.994) (Liang et al., 2023).

### 2.4 Fourier transform infrared (FT-IR) spectroscopy analysis

The SM05 sample and potassium bromide were accurately weighed at 2 mg and 200 mg, respectively. Subsequently, the SM05 sample was mixed with potassium bromide and pressed into slices. Additionally, slices of pure potassium bromide powder were prepared as blank controls. The samples were then subjected to scanning and recording using a Fourier transform infrared spectrometer FT-IR650 (Tianjin Gangdong Technology Development Co., Ltd.) (Zhang et al., 2019).

### 2.5 Scanning electron microscopy (SEM) analysis

The structure of SM05 was examined by scanning electron microscopy (SEM). Approximately 5 mg of the dried SM05 sample was mounted on a conductive carbon film using double-sided adhesive tape. The mounted sample was then placed in the sample chamber of an ion sputter coater and coated with a thin layer of gold for 40 s. The coated sample was then transferred to the SEM sample chamber. Observations were conducted at an acceleration voltage of 5 kV (Wei et al., 2022).

### 2.6 Monosaccharide composition

The monosaccharide composition of SM05 was determined by high performance liquid chromatography (HPLC). Briefly, 2 mg of SM05 was hydrolyzed with 4 mL of 2 M trifluoroacetic acid (TFA) at 110 °C for 4 h. The TFA in the sample was removed by rotary evaporation, and the residue was dissolved in 200 μL of distilled water. The aqueous solution was derivatized by adding 0.6 M NaOH and 0.5 M PMP (in methanol). The mixture was incubated at 70 °C for 100 min. After cooling, the reaction was neutralized with 0.3 M HCl. The derivatized products were then extracted three times with an equal volume of chloroform. The aqueous layer was collected and filtered through a 0.45 μm nylon membrane filter prior to HPLC injection. Detection and analysis were performed on the Hypersil BDS-C18 column (4.6 × 150 mm, 5 μm) at 40 °C. The mobile phase consisted of a mixture of phosphate buffer and acetonitrile. The flow rate was 0.8 mL/min, the injection volume was 25 μL, and isocratic elution was performed for 60 min. The following compounds were employed as reference standards: mannose, rhamnose, glucuronate, galacturonic acid, glucose, galactose, arabinose and fucose (Li et al., 2020).

### 2.7 Anti-inflammatory activity of the SM05

#### 2.7.1 Animals and treatment

The study utilized a total of 48 male C57BL/6 mice (6–8 weeks old, weighing between 18–20 g), which were procured from the Guangdong Provincial Laboratory Animal Center. All mice were kept in a SPF animal house, where the light and darkness cycle were simulated for 12 h once. The temperature of the animal house was controlled at a constant temperature of about 25 °C. All animal studies were conducted in accordance with the ARRIVE guidelines and were approved by the Laboratory Animal Ethics Committee of Zhongshan Hospital of Traditional Chinese Medicine (Certificate No. SYXK(Yue)2020-0109, Ethics Approval No. 2023043).

After 1 week of adaptive feeding, all mice were randomly divided into six groups, namely, Control, Model, SASP, SM05-L, SM05-M and SM05-H groups. The Control group was given free access to pure water for 7 days. The experimental groups, excluding the Control group, were provided with 2.0% DSS (MP Biomedicals, Irvine, CA, Cat. No. 160110) for *ad libitum* consumption over a period of seven consecutive days to induce the UC model. SASP group mice were administered SASP (MedChemExpress, NJ, United States, Cat. No. HY-14655) daily via oral gavage at a dose of 200 mg/kg throughout the experimental period. Meanwhile, SM05-L, SM05-M, and SM05-H groups received SM05 via oral gavage at daily doses of 50, 100, and 200 mg/kg, respectively. During the experimental procedures, mice were anesthetized via inhalation of isoflurane (4% for induction and 2.5% for maintenance), followed by blood collection from the orbital venous plexus (0.3–0.5 mL). Upon completion of blood sampling, euthanasia was immediately performed by cervical dislocation while the animals were still under deep anesthesia. The heart, liver, spleen, lungs, kidneys and thymus of the mice were weighed, embedded and frozen for preservation. The colon tissues of each group of mice were measured in length. The 1 cm section close to the anus was fixed and embedded for tissue, and the remaining part was stored at −80 °C for further analysis.

#### 2.7.2 Disease activity index (DAI) of UC

The daily monitoring encompassed the assessment of mice’s weight gain, presence of fecal hemorrhage, and measurement of fecal viscosity. The DAI was assessed based on the severity, and the specific scoring methodology is presented in Supplementary Table S1. In brief, as the severity of UC-related symptoms increases, the DAI score increases, with the highest score being 4 (Jeon et al., 2016).

#### 2.7.3 Histology analyses

After dissection, distal colon tissue segments were fixed in 4% paraformaldehyde for 24 h, followed by routine dehydration and paraffin embedding. Tissue sections were cut and subjected to hematoxylin and eosin (H&E) staining. Briefly, sections were deparaffinized, rehydrated, and stained with hematoxylin for 3–5 min. After rinsing with distilled water, they were differentiated in 1% acid alcohol, rinsed again, and then blued in saturated lithium carbonate solution. Subsequently, sections were dehydrated in 95% ethanol for 1 min and counterstained with eosin for 15–30 s. Finally, sections were dehydrated through a graded ethanol series, cleared in xylene, and mounted with a neutral resin. Stained sections were examined and imaged under a microscope (Nikon ECLIPSE E100, Japan) (Li et al., 2020).

#### 2.7.4 Immunohistochemistry

For immunohistochemistry (IHC), paraffin-embedded distal colon tissue was cut into 5 μm sections. Following dewaxing and rehydration, antigen retrieval was performed. The sections were then treated with 3% hydrogen peroxide in methanol for 10–15 min to quench endogenous peroxidase activity. Then, the sections were incubated with 3% BSA for 30 min to block non-specific binding sites. After that, the sections were incubated with anti-MUC2 (Proteintech, Wuhan, China, Cat No.27675-1-AP), Nrf2 (Proteintech, Wuhan, China, Cat No.16396-1-AP), HO-1(Proteintech, Wuhan, China, Cat No.10701-1-AP), NQO1(Proteintech, Wuhan, China, Cat No.11451-1-AP), Keap1(Proteintech, Wuhan, China, Cat No.10503-2-AP), Caspase-1(Proteintech, Wuhan, China, Cat No.22915-1-AP), GSDMD (Proteintech, Wuhan, China, Cat No. 20770-1-AP), NLRP3(Proteintech, Wuhan, China, Cat No.30109-1-AP) and ASC (Proteintech, Wuhan, China, Cat No.10500-1-AP) antibodies at 4 °C overnight, and then incubated with HRP secondary antibody for 50 min. The sections were developed with DAB buffer and re-stained with hematoxylin (Han et al., 2023).

#### 2.7.5 Elisa detection of inflammatory cytokines

A PBS solution containing phosphatase and protease inhibitors was prepared at a 1:50 ratio. Then, 30 mg of colon tissue was weighed and mixed with 10 volumes (v/w) of the PBS solution. Two 3-mm steel beads were added, and the mixture was ground at 80 Hz for 90 s at 4 °C, followed by incubation on ice for 40 min. Finally, the sample was centrifuged at 11,292×g for 15 min at 4 °C using a refrigerated high-speed centrifuge, and the supernatant was collected as the colon tissue homogenate. The concentrations of TNF-α (Cat. No. 370210802), IL-1β (Cat. No. 569210802), IL-6 (Cat. No. 385210802), and IL-10 (Cat. No. 371220426) were determined according to the manufacturer’s instructions. All ELISA kits were from Tianjin Annuo Ruikang Biotechnology Co., Ltd. (Tianjin, China). The expression levels of each inflammatory cytokine were calculated based on the measured protein concentrations.

#### 2.7.6 Determination of SOD activity

Tissue homogenate samples were assayed for SOD activity according to the instructions of the SOD assay kit (Nanjing Jiancheng Bioengineering Institute, China, Cat No. A001-3).

#### 2.7.7 Western blot analysis

The colon tissue of each group of mice was dissected, weighed, and homogenized with RIPA (Beyotime Biotechnology, Shanghai, China, Cat. No. P0013B) buffer at a ratio of 1:10. The resulting tissue homogenate was centrifuged, and the supernatant was collected. The protein concentration of the supernatant was determined using a BCA kit (Thermo Fisher Scientific, MA, United States, Cat. No. 23225). Based on the measured concentrations, the supernatants were diluted with PBS to a final concentration of 4 μg/μL. The diluted samples were then boiled at 100 °C for 10 min using a heat block. The electrophoresis procedure was conducted using an 8% SDS polyacrylamide gel, followed by transfer onto a PVDF membrane (Millipore, MA, United States, Cat. No. IPVH00005). After being blocked with the QuickBlock™ Blocking Buffer (Beyotime Biotechnology, Shanghai, China, Cat. No. P0235), the membrane was treated with antibodies against β-actin (1:5,000, Proteintech, Wuhan, China, Cat. No. 81115-1-RR, RRID: AB_2923704), Occludin (1:5,000, Proteintech, Wuhan, China, Cat. No. 66378-1-Ig, RRID: AB_2881755), Claudin-1 (1:5,000, Proteintech, Wuhan, China, Cat. No. 13050-1-AP, RRID: AB_2079881), and ZO-1 (1:1,000, Abcam, Cambridge, United Kingdom, Cat. No. Ab276131, RRID: AB_3083081) for 12 h at 4 °C. Then was incubated the Anti-rabbit IgG (1:5,000, Proteintech, Wuhan, China, Cat. No. SA00001-2, RRID: AB-2722564) for 2 h. The protein bands were analyzed using the ChemiDoc MP imaging system (Bio-Rad, CA, United States). Gray values were quantified using ImageJ software (Version 1.53k, NIH, United States), with β-actin serving as the loading control.

#### 2.7.8 RNA extraction and real-time quantitative polymerase chain reaction (RT-qPCR)

The RNA was isolated from colon tissue using TRIzol reagent (Thermo Fisher Scientific, MA, USA, Cat. No. 15596018). The RNA was then reverse transcribed into cDNA using PrimeScript™ RT reagent Kit (Takara, Kyoto, Japan, Cat. No. RR036A) and RT-qPCR was performed according to the manufacturer’s instructions for SYBR Green Master Mix (Takara, Kusatsu, Japan, Cat. No. RR420A). The primer sequences of Nrf2, HO-1, NQO1, Keap1, Caspase-1, IL-1β, GSDMD, NLRP3 and ASC are shown in Supplementary Table S2. The relative mRNA expression levels of the genes were calculated by the 2^(ΔΔCt) method based on the expression level of β-actin (Pan et al., 2022).

#### 2.7.9 16s rDNA sequencing

Prior to sacrifice, fresh fecal samples were collected from mice in each group and immediately stored at −80 °C. Genomic DNA was extracted from colon contents using the TIANamp fecal DNA kit. The DNA quality was measured by 0.8% agarose gel electrophoresis, and DNA was quantified by Nanodrop spectrophotometer (Thermo Scientific, New York, USA). The forward (5′-ACT​CCT​ACG​GGA​GGC​AGC​AG-3′) and reverse primers (5′-GGACTACHVGGGTWTCTAAT-3′) were used to amplify the V3∼V4 hypervariable region of the microbiome 16S rRNA gene by PCR. The PCR amplification products were detected by 2% agarose gel electrophoresis, and target fragments were recovered by gel cleavage using a DNA gel extraction kit (Axygen, New York, USA). All PCR reactions were performed using Phusion® High-Fidelity PCR Master Mix. Sequencing libraries were constructed using the Illumina TruSeq® DNA PCR-Free Sample Preparation Kit (Illumina, San Diego, CA, USA) following the manufacturer’s instructions, with the addition of unique indexing barcodes for each sample. Finally, the library was sequenced on the Illumina HiSeq 2,500 platform and a paired end reading of 250 bp was generated (Li et al., 2023).

### 2.8 Statistical analysis

When the sample size in each group was greater than 5 (n > 5), normality was assessed using the Shapiro–Wilk test, which is appropriate for small sample quantitative data. For groups with n > 5, if all data followed a normal distribution, one-way ANOVA was performed. When homogeneity of variances was satisfied, the F test was used for overall mean comparison, followed by the Student–Newman–Keuls (SNK) or Dunnett’s T3 test for pairwise comparisons. If variances were unequal, Welch’s ANOVA was applied to compare overall means, and Dunnett’s T3 test was used for multiple comparisons. When n > 5 but the data did not follow a normal distribution, or when n ≤ 5, the Kruskal–Wallis test was used to compare overall medians, followed by pairwise comparisons using the Mann–Whitney U test with Bonferroni correction. The significance level was set at α = 0.05, and a P value < 0.05 was considered statistically significant. Statistical analyses were performed using SPSS version 27, and graphs were generated using GraphPad Prism 9.0.

## 3 Results

### 3.1 Structural characterization of SM05

**FIGURE 1:**
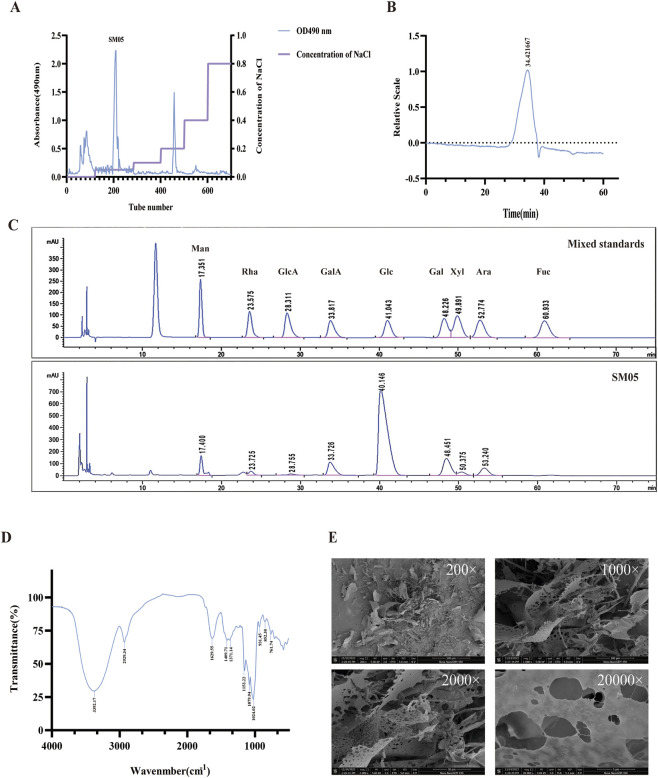
Structural characterization of SM05**. (A)** The elution curve of SM-W on the DEAE Sepharose™ Fast Flow. **(B)** Molecular weight analysis of SM05. **(C)** HPLC profiles for SM05 monosaccharide composition analysis. **(D)** The FT-IR spectra analysis of SM05. **(E)** SEM photographs of SM05.

During the extraction process, the final yields of the crude polysaccharides SMW was 2.48%. After purification by loading DEAE Sepharose fast flow column chromatography, the major fractions (named SM05) were collected from the crude polysaccharides, the yield is 3.51% of SMW (Figure 1A). The results of physicochemical property tests showed that the neutral sugar content of SM05 was determined to be 89.76%. In addition, SM05 contained a small amount of protein (9.58%) and uronic acid (5.13%).

The relative weight-average molecular weight of SM05 was 6.432 kDa (Figure 1B). The monosaccharide composition of SM05 was mannose, glucose, galactose, and arabinose with the molar ratios of 2.53%, 81.07%, 9.40% and 5.51%, respectively (Figure 1C).

### 3.2 FT-IR spectra of SM05

The FT-IR spectrum of SM05 is presented in Figure 1D. The absorption peak at 3,392 cm^-1^ is caused by the stretching vibration of O-H functional group in SM05. The absorption peak at 2,929 cm^-1^ is related to the stretching vibration of C-H in the sugar ring (Prabahar et al., 2023). Considering that SM05 contains a small amount of protein, the absorption peak at 1,629 cm^-1^ corresponds to the bending vibration of -CONH_2_ or -NH_2_. (Ismail et al., 2023). The presence of a prominent absorption peak at 1,409 cm^-1^ suggests the inclusion of -COOH functional groups in SM05 (Banerjee et al., 2014). The absorption peak observed at 1,371 cm^-1^ corresponds to the symmetric stretching vibration of the C-O bond (Nkwaju et al., 2023). The absorption peaks at 1,153 cm^-1^, 1,024 cm^-1^ are the stretching vibrations of C-O-C and C-O-H on the pyran ring (Zhu et al., 2023). The absorption peak at 931 cm^-1^ is the asymmetric stretching vibration of the C-O-C skeleton of D-glucosyl pyran (Ouyang et al., 2021). The absorption peak at 851 cm^-1^ indicates that the glycosidic bond is α-type (Xiong et al., 2023). The absorption peak at 761 cm^-1^ is the C-O-C vibration of the D-glucosyl pyran ring (Ouyang et al., 2021).

### 3.3 SEM analysis of SM05

In SEM images (Figure 1E), SM05 showed a porous sheet, relatively thin, and part of it was striped. The voids on the laminates are round or oval, arranged at irregular intervals, with smooth chromatographic edges and clear boundaries. The surface is scaly attached to the surface, the round hole part is branched, continuous or broken, and the size of the round hole is different.

### 3.4 SM05 alleviated the symptoms in DSS-induced UC mice

**FIGURE 2:**
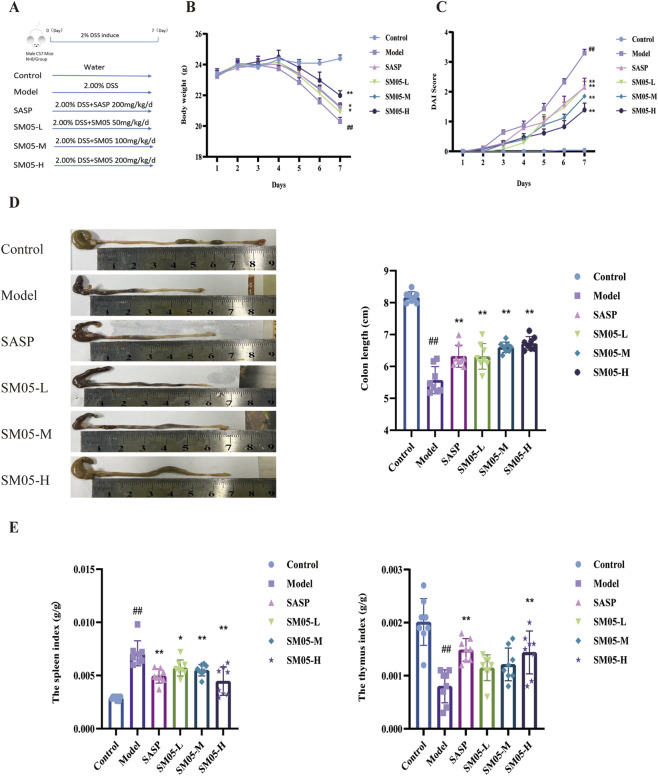
Effects of SM05 in mice subjected to DSS-induced colitis. **(A)** Experimental arrangement and animal grouping. **(B)** Bodyweight changes in each group (n = 8). **(C)** DAI scores in each group (n = 8). **(D)** Corresponding representative images of colon of each group of mice and statistical analysis of colon length of each group (n = 8). **(E)** The spleen index and the thymus index of each group (n = 8). Data are presented as means ± S.E.M. Different letters indicate significant differences (*P* < 0.05) among the six groups. ^#^ *P* < 0.05, ^##^ *P* < 0.01 vs. Control group; ^*^ *P* < 0.05, ^**^ *P* < 0.01 vs. Model group.

The C57BL/6 mice were given free DSS for 7 consecutive days to induce UC (Figure 2A). During the modeling period, compared with the Control group, Model group mice showed decreased body weight (Figure 2B) and an increase in DAI scores (Figure 2C). After the experiment, the colons of each group of mice were collected and their lengths were measured. The colons of DSS group mice were significantly shortened compared to the Control group (Figure 2D). SM05 and SASP treatment can alleviate these adverse reactions. In addition, the abnormal changes of spleen index and thymus index can be restored to a certain extent after SM05 treatment (Figure 2E).

### 3.5 SM05 maintained the integrity of mucosal barrier in DSS-induced UC mice

**FIGURE 3:**
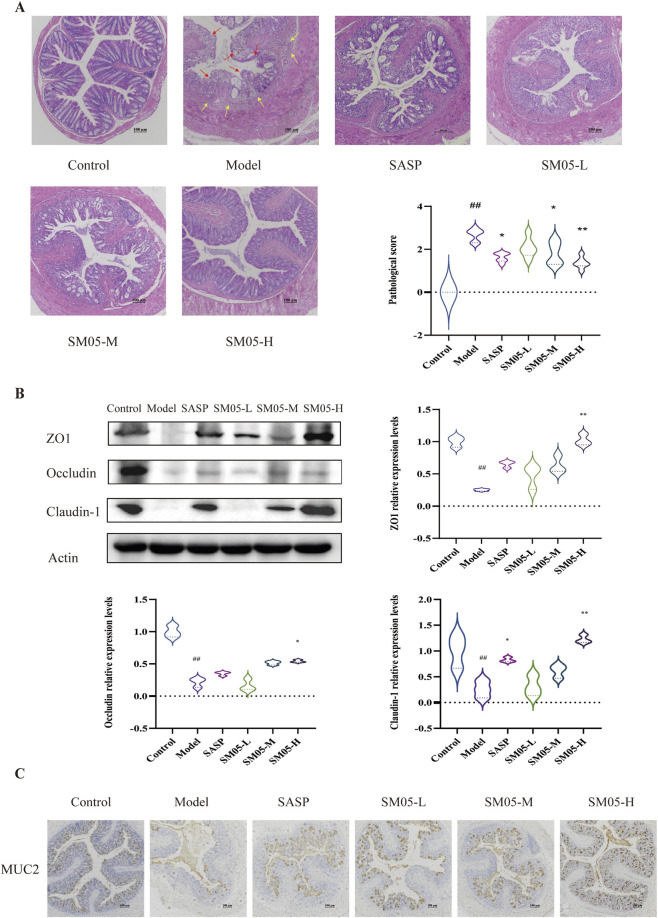
SM05 repaired intestinal barrier function in DSS-induced UC mice. **(A)** Representative H&E staining images and Histopathological score (n = 6). Yellow arrow: inflammatory cells; red arrow: intestinal villi damage. **(B)** The level of ZO1, Occludin and Claudin-1 in colonic tissues by Western blot (n = 3). **(C)** IHC analysis of MUC2 protein detection in colon tissue (n = 3). All images are at ×100 magnification. Data are presented as the means ± S.E.M. Different letters indicate significant differences (*P* < 0.05) among the six groups. ^#^ *P* < 0.05, ^##^ *P* < 0.01 vs. Control group; ^*^ *P* < 0.05, ^**^ *P* < 0.01 vs. Model group.

Compared with the Control group, Model group mice exhibited significant tissue inflammation and severe histological injury (Figure 3A), characterized by decreased goblet cells, increased inflammatory infiltration, epithelial erosion, crypt architectural distortion, and submucosal edema. A certain dose of SM05 can protect the microstructure of the colon and reduce intestinal tissue damage caused by DSS. In addition, oral administration of SM05 significantly maintained the colonic mucosal barrier through varying degrees of upregulation of Occludin, Claudin-1, and ZO-1 expression (Figure 3B).

The mucin 2 (MUC2) secreted by goblet cells in colon tissue is the functional basis of the mucus barrier (Bourragat et al., 2024). Immunohistochemical analysis of MUC2 in colon tissues of mice in each group showed that compared with the normal group, the expression of MUC2 was inhibited in the Model group, and the expression of MUC2 was significantly upregulated after administration of SM05. In general, these results suggest that SM05 can maintain the integrity of the mucosal barrier in mice with DSS induced colitis (Figure 3C).

### 3.6 SM05 regulate the levels of inflammatory cytokines in DSS induced UC mice

**FIGURE 4:**
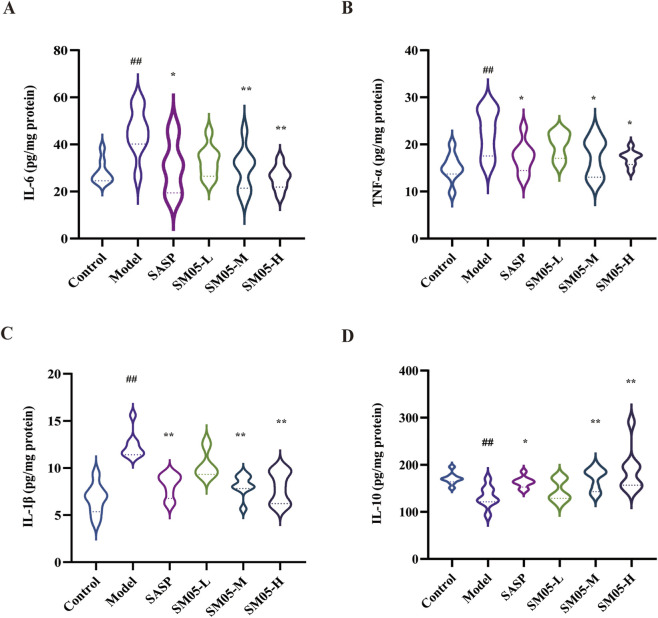
SM05 regulated the colon inflammatory cytokines level in DSS-induced UC mice. The levels of **(A)** IL-6, **(B)** TNF-α, **(C)** IL-1β, **(D)** IL-10 in mice colons (n = 8). Different letters indicate significant differences (*P* < 0.05) among the six groups. ^#^ *P* < 0.05, ^##^ *P* < 0.01 vs. Control group; ^*^ *P* < 0.05, ^**^ *P* < 0.01 vs. Model group.

In this study, the pro-inflammatory cytokines IL-1β, IL-6 and TNF-α were significantly increased in colon tissue of mice in the Model group compared with the Control group. Treatment with SM05 reduce the levels of inflammatory cytokines in DSS induced UC mice (Figures 4A–C). The experimental results showed that compared with the normal group, the anti-inflammatory cytokines IL-10 level in the colon tissue of the Model group mice was significantly reduced. After treatment with SM05, IL-10 levels can be significantly increased and even restored to normal levels (Figure 4D). The result now provides evidence that SM05 can regulate the imbalance between pro-inflammatory and anti-inflammatory cytokines caused by DSS.

### 3.7 SM05 inhibits oxidative stress response in DSS induced UC mice

**FIGURE 5:**
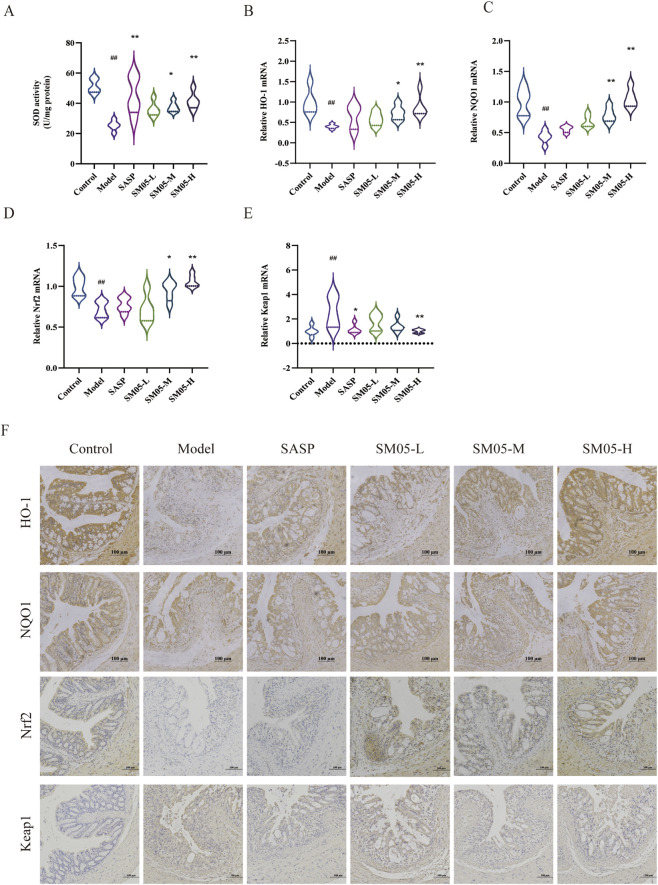
SM05 inhibits oxidative stress response in DSS-induced UC mice. **(A)** SOD activity in colon tissue (n = 5). **(B–E)** qRT-PCR detection of HO-1 **(B)**, NQO1 **(C)**, Nrf2 **(D)**, Keap1 **(E)** gene expression levels (n = 5). **(F)** IHC analysis of HO-1, NQO1, Nrf2, Keap1 protein detection in colon tissue (n = 3). All images are at ×200 magnification. Data are presented as the means ± S.E.M. Different letters indicate significant differences (*P* < 0.05) among the six groups. ^#^ *P* < 0.05, ^##^ *P* < 0.01 vs. Control group; ^*^ *P* < 0.05, ^**^ *P* < 0.01 vs. Model group.

To determine the antioxidant effect of SM05, the expression levels of Nrf2, HO-1, NQO1 and Keap1 in the colon tissue were further analyzed. The levels of these genes in the colon tissue were detected by RT-qPCR. The results showed that SM05 promoted the expression of Nrf2, HO-1 and NQO1 and inhibited Keap1 (Figures 5B–E), thereby enhancing the antioxidant activity of the colon tissue in mice. To further evaluate the effect of SM05, the expression of related proteins in the colon tissue was also detected by IHC. The IHC results showed that compared with the Model group, the levels of HO-1, NQO1 and Nrf2 in the treatment group were significantly increased, and the level of Keap1 was significantly decreased (Figure 5F).

### 3.8 SM05 inhibits the occurrence of pyroptosis in DSS induced UC mice

**FIGURE 6:**
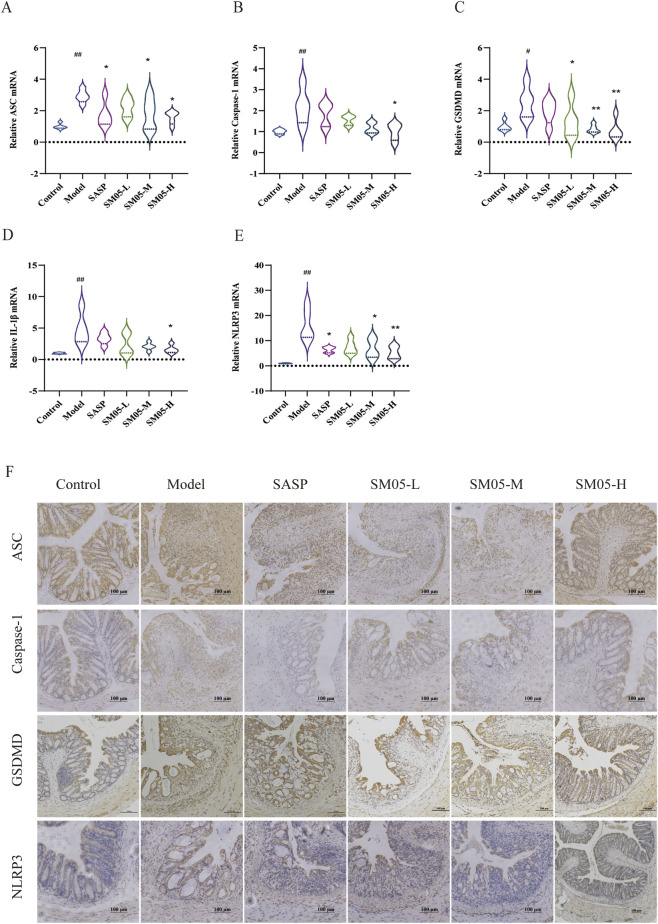
SM05 inhibits pyroptosis by regulating the NLRP3 inflammasome. **(A–E)** qRT-PCR detection of ASC **(A)**, Caspase-1 **(B)**, IL-1β **(C)**, GSDMD **(D)** and NLRP3 **(E)** gene expression levels (n = 5) **(F)** IHC analysis of ASC, Caspase-1, IL-1β, GSDMD and NLRP3 protein detection in colon tissue (n = 3). All images are at ×200 magnification. Different letters indicate significant differences (*P* < 0.05) among the six groups. ^#^ *P* < 0.05, ^##^ *P* < 0.01 vs. Control group; ^*^ *P* < 0.05, ^**^ *P* < 0.01 vs. Model group.

As shown in Figure 5A, administration of SM05 significantly increased SOD activity in the colonic tissue of UC mice, suggesting an enhancement of the endogenous antioxidant defense system. In order to investigate whether SM05 exerts its anti-UC effect by inhibiting cell pyroptosis, we detected the expression of pyroptosis related genes. As shown in Figures 6A–E, compared with the Control group, the mRNA expressions of NLRP3, ASC, IL-1β, Caspase-1 and GSDMD in the Model group were significantly increased. However, these changes were significantly reduced in the SM05 administration group. DSS induced activation of the NLRP3 inflammasome signaling pathway led to a significant increase in IL-1β levels, while SM05 inhibited its increase in colon tissue. The results confirmed that oral administration of SM05 significantly blocked activation of the NLRP3 inflammasome pathway induced by DSS. The protein expression levels of NLRP3, ASC, Caspase1 and GSDMD in the colon of DSS treated mice were prominently increased, while SM05 effectively inhibited the increase of these protein expressions, suggesting that SM05 can affect the NLRP3 inflammasome pathway at the protein level (Figure 6F).

### 3.9 SM05 remodeled the gut microbiota structure in DSS-induced UC mice

**FIGURE 7:**
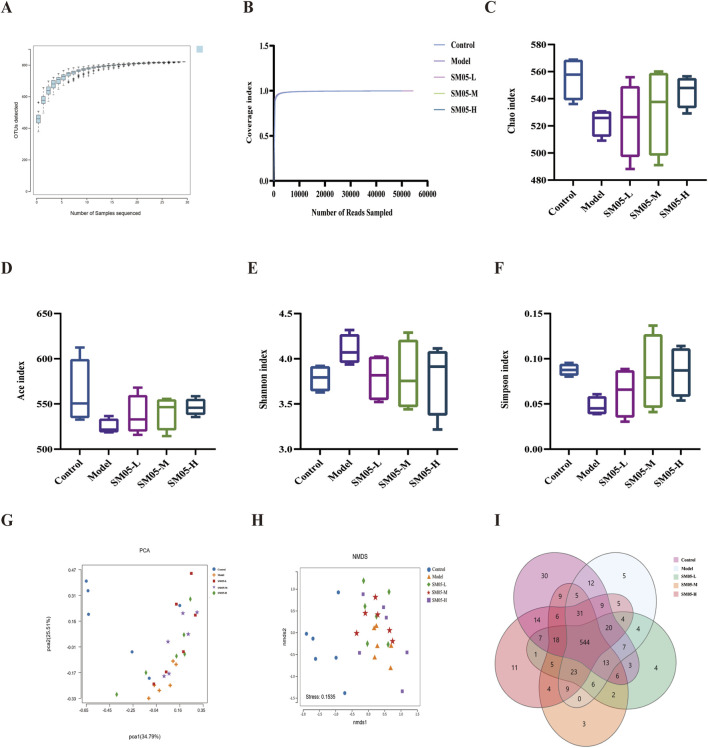
SM05 remodeled the gut microbiota structure in DSS-induced colitis mice. **(A)** Species accumulation curve. **(B)** Coverage index. **(C–F)** The alpha diversity includes **(C)** Chao index, **(D)** Ace index, **(E)** Shannon index and **(F)** Simpson index (n = 4). **(G)** NMDS analysis (n = 6). **(H)** PCA analysis (n = 6). **(I)** A Venn diagram between six groups (n = 6). Different letters indicate significant differences (*P* < 0.05) among the six groups. ^#^ *P* < 0.05, ^##^ *P* < 0.01 vs. Control group; ^*^ *P* < 0.05, ^**^ *P* < 0.01 vs. Model group.

In this study, 16s rDNA sequencing was used to determine the effect of SM05 on the gut bacteria of mice with DSS induced UC. As the amount of sequencing increases, the observed species accumulation curve gradually stabilizes, with a coverage index above 0.99, indicating that the sequencing data is sufficiently reliable for further analysis (Figures 7A,B). The results of α diversity analysis showed that DSS induction reduced the species abundance and increased the species richness of the ecosystem of mice. Supplementing SM05 can reverse the changes induced by DSS (Figures 7C–F). PCA and NMDS results showed a significant separation between the Control and Model groups, indicating that DSS altered the overall structure of the gut microbiota. However, treatment with SM05 remodeled the gut microbiota structure disturbed by DSS, indicating that SM05 were conducive to remodeling of the gut microbiota structure in mice with DSS-induced colitis (Figures 7G,H). The Venn diagram based on OTU statistics shows the number of common and specific species in each group of mice. Results showed that the Control, Model, SM05-L, SM05-M and SM05-H groups shared 544 OTUs in total and own 30, 5, 4, 3 and 11 unique OTUs, respectively (Figure 7I).

### 3.10 SM05 regulates the composition and abundance of gut microbiota in DSS-induced UC mice

**FIGURE 8:**
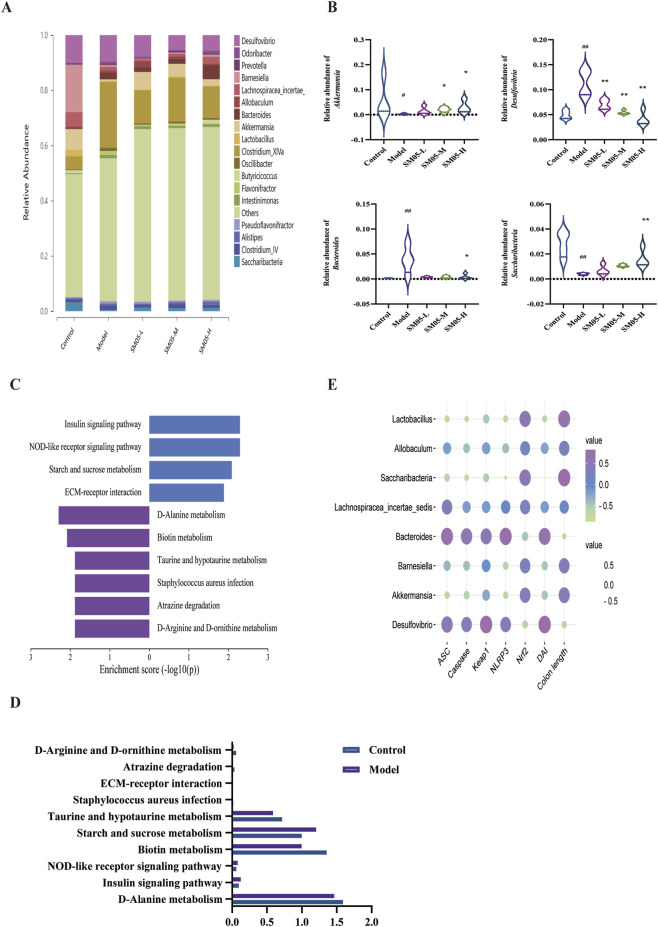
SM05 regulates the composition and abundance of gut microbiota in mice. **(A)** Relative abundance of gut microbiota in species level. **(B)** Relative abundance expression of Akkermansia, *Bacteroides*, Desulfovibrio, Saccharibacteria. **(C,D)** Functional difference analysis in level 3 includes **(C)** the comparison between Control and Model enrichment score, **(D)** the comparison between Control and Model Relative Abundance. **(E)** Balloonplot of Spearman correlation analysis between 11 most differential genera and biochemical parameters (n = 4). Different letters indicate significant differences (*P* < 0.05) among the six groups ^#^ *P* < 0.05, ^##^ *P* < 0.01 vs. Control group; ^*^ *P* < 0.05, ^**^ *P* < 0.01 vs. Model group.

At the species level, 18 major groups of gut microbiota were identified (Figure 8A). Compared with the Control group, *Akkermansia* in the intestine of mice induced by DSS was significantly reduced, and SM05 could improve this phenomenon to a certain extent. Similarly, administration of DSS also reduced the level of *Saccharibacteria* in the gut of mice, and administration of SM05 changed this symptom. In addition, the relative abundance of pathogenic bacteria such as *Bacteroides*, and *Desulfovibrio*in the gut of DSS treated mice were significant increased. After DSS induction, the relative abundance of *Bacteroides* and *Desulfovibrio* in the intestines of mice were significantly increased while SM05 significantly reduced it. These results indicated that SM05 could adjust the microflora structure in DSS induced UC mice, increase the relative abundance of beneficial bacteria and decrease the relative abundance of harmful bacteria (Figure 8B).

Dysregulation of gut bacteria in UC mice induced by DSS may be accompanied by functional disruption. The gut microbiome induced metabolic function was compared according to the classification composition of Control and Model treatments by KEGG functional pathway prediction. The results showed that DSS could further activation of insulin signaling pathway, NOD-like receptor signaling pathway, starch and sucrose metabolism signaling pathway (Figures 8C,D). At the same time, DSS inhibited D-alanine metabolism, biotin metabolism, taurine and low taurine metabolism, D-arginine and D-ornithine metabolism and other metabolic pathways (Figures 8C,D). Through meticulous correlation analysis, it was revealed that crucial alterations in the intestinal microbiota were intimately associated with the NLRP3-induced pyroptosis pathway (Figure 8E).

## 4 Discussion

As a commonly used traditional Chinese herbal medicine, *C. foetida* L. is often utilized in the clinical treatment of UC. However, the primary active constituents and the precise mechanism of action of *C. foetida* L. in treating UC remain underexplored in current research. Therefore, in the present study, the polysaccharide subfraction SM05 with anti-UC activity was isolated from dried *C. foetida* L. and subjected to a preliminary biological evaluation as a partially characterized fraction.

After structural analysis, SM05 was found to be a heteropolysaccharide composed of glucose, mannose, galactose and arabinose, of which glucose accounted for a relatively high content. The monosaccharide composition of SM05 may have a beneficial effect on intestinal barrier protection (Liu et al., 2023). For example, it has been reported that Astragalus polysaccharides composed of Gal, Ara, and Man monosaccharides can protect the intestinal barrier and alleviate the development of UC by inhibiting the NLRP3 inflammasome (Tian et al., 2017). The smaller molecular weight of SM05 may confer it with better reductive properties (Shi et al., 2013). Additionally, the overall loose and porous structure of SM05 increase its specific surface area, allowing for better biological activity (Zhang et al., 2023). It is worth noting that the SM05 extracted in this study remains a complex mixture, containing a certain amount of protein and uronic acid. To elucidate the relationship between its structure and activity, subsequent isolation of homogeneous polysaccharides from SM05 is required, along with more in-depth structural analysis.

Through *in vivo* animal experiments, it was confirmed that SM05 can alleviate the symptoms of weight loss, DAI score increase, and decreased colon length induced by DSS in UC mice. Meanwhile, SM05 exhibited good intestinal barrier protection, maintaining the integrity of the intestinal barrier in UC mice. Furthermore, SM05 can also inhibit the secretion of pro-inflammatory cytokines TNF-α, IL-6, and IL-1β in the colon tissue of UC mice, and promote the increase of anti-inflammatory cytokine IL-10. These results indicate that SM05 exhibits significant anti-UC activity and has potential therapeutic value.

NLRP3 inflammasome is a cytosolic protein complex that can recognize both exogenous pathogens and endogenous danger signals (Lu et al., 2023). The excessive activation of NLRP3 can induce Caspase-1-dependent cell pyroptosis (Zhang et al., 2022). The abnormal occurrence of pyroptosis leads to cell expansion and rupture, resulting in the secretion of IL-1 β and IL-18 into the extracellular space (Li et al., 2022). IL-1β and IL-18 recruit additional immune inflammatory cells, further amplifying the inflammatory response, leading to the disruption of the intestinal mucosal barrier and promoting the development of UC (Han et al., 2022). The activation of the pyroptosis pathway may be associated with the induction of a substantial quantity of peroxides (Ferrada et al., 2022). Nrf2/Keap1 pathway is considered to be the basic mechanism of coordinating cellular antioxidant response, and has become an important target for the prevention and treatment of various inflammatory diseases (Mei et al., 2019). It is noteworthy that the Nrf2/Keap1 pathway has been demonstrated to exhibit a close association with pyroptosis. Fu, F et al. showed that pyroptosis could be inhibited by activating the Nrf2/Keap1 pathway (Fu et al., 2023). This study results demonstrated that SM05 effectively induced the upregulation of genes and proteins associated with the Nrf2/Keap1 antioxidant pathway, while concurrently suppressing the NLRP3-mediated pyroptosis pathway.

Due to the lack of genes encoding related digestive enzymes in the human genome, most of the TCM polysaccharides cannot be directly digested and absorbed by the human body after oral administration. However, the microorganisms in the human intestine can encode a variety of polysaccharide digestive enzymes, which can digest most of the heteropolysaccharides (Liu et al., 2022). TCM polysaccharides can reshape the structure of intestinal flora, significantly increase the abundance of beneficial bacteria, and inhibit pathogenic bacteria.

Simultaneously, the intestinal microbiota structure of each group of mice was analyzed using 16s rDNA sequencing. The results showed that SM05 could increase the abundance of beneficial bacteria in the intestine of DSS-induced colitis mice and decrease the abundance of pathogenic bacteria. The NOD-like response signaling pathway was found to be activated in DSS-induced colitis mice through functional analysis using KEGG, which is consistent with previous animal experiments.

*Akkermansia*, *Bacteroides*, *Desulfovibrio*, and *Saccharibacteria* are likely to exert a significant influence on this process. The study conducted by Jennings-Almeida et al. has demonstrated the inhibitory effect of *Akkermansia* on NLRP3 mRNA expression. *Bacteroides* and *Desulfovibrio* were found to promote NLRP3-induced pyroptosis (Jennings-Almeida et al., 2021; An et al., 2023). The association between *Saccharibacteria* and the pathogenesis of UC remains inadequately investigated. Interestingly, there was a significant negative correlation between the relative abundance of *Saccharibacteria* in the gut and UC across various experimental studies.

In summary, this study provides a preliminary investigation into the structure of Cimicifuga polysaccharides and their pharmacological mechanisms against ulcerative colitis. However, since only secondary polysaccharides were isolated and purified, and no homogeneous polysaccharide has been obtained, there remain certain limitations in the structural characterization. Furthermore, although our study reveals that SM05 can regulate the Nrf2/Keap1 pathway and the NLRP3-induced pyroptosis pathway, whether there is a direct interaction between these two pathways requires further experimental validation. In addition, the relationship between NLRP3-mediated pyroptosis and the gut microbiota has not been verified by germ-depleting or fecal microbiota transplantation experiments. These limitations could serve as directions for further extension of the current study.

## 5 Conclusion

The secondary polysaccharide subfraction SM05 extracted from *C. foetida* L. exhibits excellent anti-UC activity. Its mechanism of action is likely associated with the inhibition of the pyroptosis pathway induced by NLRP3 and the modulation of the balance of gut microbiota.

## Data Availability

The original contributions presented in the study are publicly available. This data can be found here: https://www.ncbi.nlm.nih.gov/bioproject/PRJNA1476895.

## Glossary

ASC: Apoptosis-associated speck-like protein containing a CARD
BCA: Bicinchoninic Acid Assay
DAI: Disease Activity Index
DSS: Dextran Sulfate Sodium
FT-IR: Fourier Transform Infrared Spectroscopy
GSDMD: Gasdermin D
HPLC: High Performance Liquid Chromatography
HO-1: Heme Oxygenase-1
HPGPC: High Performance Gel Permeation Chromatography
IL-1β: Interleukin-1 beta
IL-6: Interleukin-6
IL-10: Interleukin-10
IL-18: Interleukin-18
IHC: Immunohistochemistry
Keap1: Kelch-like ECH-associated protein 1
KEGG: Kyoto Encyclopedia of Genes and Genomes
MUC2: Mucin 2
NLRP3: NOD-like receptor family pyrin domain containing 3
NOD-like: Nucleotide-binding Oligomerization Domain-like receptors
NQO1: NAD(P)H Quinone Oxidoreductase 1
Nrf2: Nuclear factor erythroid 2-related factor 2
NMDS: Non-metric Multidimensional Scaling
OTU: Operational Taxonomic Unit
RT-qPCR: Reverse Transcription Quantitative Polymerase Chain Reaction
SASP: sulfasalazine
SEM: Scanning Electron Microscope
SPF: Specific Pathogen Free
TCM: Traditional Chinese Medicine
TNF-α: Tumor Necrosis Factor alpha
UC: Ulcerative Colitis
ZO-1: Zonula Occludens-1
