# Supplementary material for: Cimicifuga foetida L. polysaccharide alleviates ulcerative colitis by inhibiting pyroptosis and regulating gut microbiota
Source: Front Pharmacol. 2026 Jul 13;17:1780518. doi: 10.3389/fphar.2026.1780518 (PMC13402411; doi:10.3389/fphar.2026.1780518)
Supplement: Supplementary file 1 [file Table1.docx]

**Table 1 Disease activity index (DAI) evaluation criteria**

| Score | Weight Loss (%) | | Stool Consistency | Occult/Gross Bleeding |
| --- | --- | --- | --- | --- |
| 0 | <1 | | Normal | Normal |
| 1 | 1~5 |  | |  |
| 2 | 6~10 | | Loose stool | Positive |
| 3 | 11~15 | |  |  |
| 4 | >15 | | Diarrhea | Gross bleeding |

**Table 2 Primer sequences used for RT-qPCR analysis**

| Gene Name |  | Sequence (5'-3') |
| --- | --- | --- |
| GSDMD | Forward | 5'-CGGAAAGATTTTACAGGACCAGCC-3' |
|  | Reverse | 5'-AGAGTCAAGCACCAGACACTCAAG-3' |
| NLRP3 | Forward | 5'-GATCAACAGGCGAGACCTCTG-3' |
|  | Reverse | 5'-CCAGCAAACCCATCCACTCTT-3' |
| ASC | Forward | 5'-TGTGCTTAGAGACATGGGCTTACA-3' |
|  | Reverse | 5'-AAAGTGTCCTGTTCTGGCTGTACT-3' |
| Caspase-1 | Forward | 5'-CTGGCAGGAATTCTGGAGCTT-3' |
|  | Reverse | 5'-AGTCAGTCCTGGAAATGTGCC-3' |
| Keap1 | Forward | 5'-CACAGCAGCGTGGAGAGATATGAG-3' |
|  | Reverse | 5'-AGTAACATTCTGCGGAGTTAAGCC-3' |
| NQO1 | Forward | 5'-TCTCTAGCATATAAGGAAGGACGCC-3' |
|  | Reverse | 5'-TATGTGTAGGCAAATCCTGCTACGA-3' |
| HO-1 | Forward | 5'-GTCAGGTGTCCAGAGAAGGCTTTA-3' |
|  | Reverse | 5'-GAAGTAGAGTGGGGCATAGACTGG-3' |
| Nrf2 | Forward | 5'-TAGAGCAGGACATGGAGCAAGTTT-3' |
|  | Reverse | 5'-CATTTCTGTCAGTGTGGCTTCTGG-3' |
| β-actin | Forward | 5'-CAACGGCTCCGGCATGTG-3' |
|  | Reverse | 5'-AGTCCTTCTGACCCATTCCCA-3' |
